# Aspirin Colorectal Cancer Prevention in Lynch Syndrome: Recommendations in the Era of Precision Medicine †

**DOI:** 10.3390/genes13030460

**Published:** 2022-03-03

**Authors:** Davide Serrano, Paola Patrignani, Vittoria Stigliano, Daniela Turchetti, Stefania Sciallero, Franco Roviello, Alessandro D’Arpino, Ignazio Grattagliano, Salvo Testa, Cristina Oliani, Lucio Bertario, Bernardo Bonanni

**Affiliations:** 1 Division of Cancer Prevention and Genetics, European Institute of Oncology IRCCS, 20141 Milan, Italy; davide.serrano@ieo.it (D.S.); bernardo.bonanni@ieo.it (B.B.); 2 Department of Neuroscience, Imaging and Clinical Sciences, and CAST, “G. d’Annunzio” University, 66100 Chieti, Italy; ppatrignani@unich.it; 3 Division of Gastroenterology and Digestive Endoscopy, IRCCS, Regina Elena National Cancer Institute, 00144 Rome, Italy; vittoria.stigliano@ifo.it; 4 Center for Hereditary Cancer, Department of Medical and Surgical Sciences, University of Bologna, 40100 Bologna, Italy; daniela.turchetti@unibo.it; 5 IRCCS Ospedale Policlinico San Martino, 16100 Genoa, Italy; stefania.sciallero@hsanmartino.it; 6 Unit of General Surgery and Surgical Oncology, Department of Medicine, Surgery and Neurosciences, University of Siena, 53100 Siena, Italy; franco.roviello@gmail.com; 7 Hospital Pharmacy Unit, Santa Maria della Misericordia Hospital, Azienda Ospedaliera di Perugia, 06100 Perugia, Italy; alessandro.darpino@ospedale.perugia.it; 8 Italian College of General Practitioners and Primary Care, 70100 Bari, Italy; studiomedico@grattagliano.it; 9 Mutagens Foundation, 20100 Milano, Italy; presidente@mutagens.it; 10 Ambulatorio Familiarita’ Neoplastica UOC Oncologia Medica ULSS5 Polesana, 45100 Rovigo, Italy; cristina.oliani@aulss5.veneto.it

**Keywords:** colorectal cancer, lynch syndrome, aspirin

## Abstract

Cancer prevention in the era of precision medicine has to consider integrated therapeutic approaches. Therapeutic cancer prevention should be offered to selected cohorts with increased cancer risk. Undoubtedly, carriers of hereditary cancer syndromes have a well-defined high cancer risk. Lynch Syndrome is one of the most frequent hereditary syndromes; it is mainly associated with colorectal cancer (CRC). Nonsteroidal anti-inflammatory drugs and, in particular, aspirin use, has been associated with reduced CRC risk in several studies, initially with contradictory results; however, longer follow-up confirmed a reduced CRC incidence and mortality. The CAPP2 study recruited 861 Lynch syndrome participants randomly assigned to 600 mg of aspirin versus placebo. Like sporadic CRCs, a significant CRC risk reduction was seen after an extended follow-up, with a median treatment time that was relatively short (2 years). The ongoing CAPP3 will address whether lower doses are equally effective. Based on pharmacology and clinical data on sporadic CRCs, the preventive effect should also be obtained with low-dose aspirin. The leading international guidelines suggest discussing with Lynch syndrome carriers the possibility of using low-dose aspirin for CRC prevention. We aim systematically promote this intervention with all Lynch syndrome carriers.

## 1. Cancer Prevention Overview

Cancer prevention for the general population is commonly associated with screening programs with early detection to lower cancer mortality as the primary endpoint. In parallel to this approach, preventive interventions strategies have been investigated to reduce cancer incidence. Therapeutic cancer prevention has great potential, but more research and educational and communication programs for health care providers and the general population are needed.

Colorectal cancer (CRC) is still among the more common cancers in terms of incidence and mortality worldwide [1]. A recent publication focusing on European countries showed significant differences in CRC mortality and incidence, between countries where screening programs were well established, compared to countries that developed them only recently or those with no screening programs [2]. Countries with more extensive screening showed a significant CRC mortality reduction. The impact of high screening coverage shows an initial increase of cancer cases found within the first and second rounds of screening and a subsequent decline. In contrast, CRC incidence was stable or increased in countries lacking screening programs.

Screening programs are designed for the general population, and, especially in countries where there is publicly funded health care, age is the only discriminant to access to the program. In the era of precision medicine, screening programs should be personalized. For instance, in a recent paper by Helsingen et al. [3], a CRC screening program based on a predictive risk model was proposed. The expert panel suggested that subjects with a cancer risk ≥3% at 15 years should undergo screening with one of the available screening options, whereas those with cancer risk <3% at 15 years might not need to be screened. Helsingen’s proposal avoids CRC screening for very low-risk populations; however, we should consider more accurate screening tests for high-risk individuals to improve effectiveness even if this is more invasive. This strategy requires an active interaction between healthcare providers and the population to identify individual risk and to reach a proper balance between health assistance and personal preferences, the potential benefits and disadvantages of the test/program offered, and to obtain a real shared and informed decision. Proper cancer risk awareness significantly improves preventive program adherence [4].

Proposing a preventive treatment with a drug, which implies possible side effects and a long treatment period, underlines the importance of stratifying people based on their own risk. Higher disease risk may justify a different risk/benefit ratio of a specific drug intervention.

Large cohort studies on cancer prevention, particularly on CRC prevention, are hampered by the time length, as they could take at least ten years before they produce evidence of the effect on cancer events. At the same time, the success of cardiovascular prevention at long-term follow-up was based on studies with reliable surrogate biomarkers. This supports the effectiveness of preventive interventions in the short–medium term. Hence the need to find and validate effective biomarkers to identify high-risk individuals better and predict the intervention efficacy in cardiovascular disease and CRC.

The primary clinical marker for CRC prevention is adenoma due to its role as a precancerous lesion, and polypectomy reduces the risk of CRC [5]. Adenoma detection and recurrence can also be evaluated to verify the efficacy of preventive drug interventions based on many lines of clinical evidence of aspirin use in CRC prevention [6]. However, a very recent meta-analysis of randomized clinical trials [7], comparing the efficacy of daily aspirin use to placebo in healthy individuals at the time of study entry, showed controversial results. A significant 22% incidence reduction for advanced lesions (i.e., adenomas with a villous component, adenomas ≥1 cm in diameter, adenomas with high-grade dysplasia, and/or invasive cancer) was seen at 5 years but not at 3 or 10 years. The subgroup analysis showed that the effect was restricted to the aspirin medium-high doses (≥300 mg/day). An opposite trend was seen for adenomas, where a significant 16% reduction was seen at 3 years with the low-dose aspirin group (≤160 mg/day). No difference was observed in adverse events. The major limitation to interpreting these data is missing information about the duration of aspirin intake. Interestingly, the positive effect for adenomas is seen early with a lower dose. For advanced lesions, it is seen at longer follow-up with higher doses. These findings suggest that low- and high-dose aspirin can affect different mechanisms of disease biology, as discussed below.

The systematic review and meta-analysis of randomized clinical trials by Veettil et al. [8] showed positive results on a population with a previous history of CRC or adenomas. Low-dose aspirin for 2–4 years significantly reduced the recurrence of any adenoma, but the data for advanced adenomas were less robust. Additionally, selective cyclooxygenase (COX)–2 inhibitors (coxibs) significantly reduced adenoma recurrence. Additionally, a trend of increased risk after quitting the drug, particularly for coxibs, was shown [8].

A recent large cancer screening trial over a median duration of 13 years [9] showed that the traditional nonsteroidal anti-inflammatory drug (NSAID) ibuprofen (≥30 versus <4 pills per month) decreased the risk of advanced distal adenoma in standard risk individuals. Aspirin was more effective on adenoma recurrence (≥30 versus <4 pills per month). Both NSAIDs showed a preventive effect on cancer incidence except for rectal cancer [9]. This study has several limitations, including missing data on (1) the NSAID use with proximal adenomas; (2) the NSAID dose taken; (3) the NSAID use information subsequent to baseline. Altogether, these points make the results hard to interpret.

## 2. Aspirin and Cancer Prevention

Many lines of clinical evidence support the potential role of aspirin in cancer prevention [6]; however, its use in clinical practice is held back by the possible risk of side effects, mainly bleeding [10]. Considering data from the last decade, the meta-analysis by Rothwell et al. has to be mentioned [11]. It included eight randomized clinical trials for primary or secondary prevention of vascular disease. Participants were randomized to aspirin versus placebo, the aspirin dose covering a wide range (from 75 to 1200 mg per day). Data showed a death reduction from different cancers starting after the fifth year of follow-up. Within the second decade, specifically for CRC, a statistical significant death reduction reached 49%, maintaining a 40% reduction with longer follow-up. For CRC, aspirin, even at the lower dose of 75 mg/day used for cardiovascular disease prevention, reduced cancer mortality and cancer incidence [12]. The incidence decreased by 24% (*p* = 0.02) for colon cancer, reaching a 55% reduction (*p* = 0.001) for proximal colon, but a not significant 10% reduction for rectal cancer was shown. The risk-reducing effect of aspirin on cancer is seen overall in the gastrointestinal tract. Further analyses of cancer events in randomized clinical trials with aspirin [11,13,14,15] for cardiovascular disease prevention showed: (1) detectable benefits at daily doses as low as 75 mg; (2) an apparent chemopreventive effect of aspirin saturable at low doses (i.e., 10- to 20-fold higher doses were not more effective than lower doses); and (3) a chemoprevention apparent effect in men at high cardiovascular risk treated with a 75-mg of controlled-release aspirin.

A recent meta-analysis of observational studies [16] showed a relative risk (RR) of 0.73 (95% CI, 0.69–0.78) for CRC; RR 0.67 (95% CI, 0.57–0.79), and RR 0.61 (95% CI, 0.0.49–0.77) respectively for squamous esophageal cancer and adenocarcinoma of esophageal and gastric cardia; RR 0.64 (95% CI, 0.51–0.82) for stomach cancer; RR 0.78 (95% CI, 0.68–0.89) and RR 0.62 (95% CI, 0.44–0.79) for pancreatic cancer and hepato-biliary tract respectively.

The risk reduction observed was similar between colon and rectal cancer. The authors showed a dose- and time-dependent linear response for CRC. In contrast to the results of the metanalyses of randomized clinical trials, a dose-response was apparently detected in this study. Out of 11 studies, a significant 10% reduction with 75 mg/day up to 50% with 500 mg per day was shown. Out of 22 studies, a time-dependent risk reduction was found, i.e., 4%, 19%, and 29% reduction were seen at one, five, and ten years of treatment, respectively [16].

Recent observational studies strengthened the positive effect of regular aspirin use to reduce CRC risk and specific mortality [17,18]. The data were stratified by pre- and post-cancer diagnosis aspirin intake to investigate the effect on cancer mortality.

Overall, pre and post intake showed a significant 30 to 40% mortality reduction. The finding was that long-term aspirin use before a diagnosis of nonmetastatic CRC may be associated with lower CRC-specific mortality after diagnosis, consistent with possible inhibition of micrometastases before diagnosis. Recently, Lau et al. [19] showed that standard cardiovascular disease risk factors are associated with increased risk of future cancer in participants (free of cancer at baseline) of FHS (Framingham Heart Study) and PREVEND (Prevention of Renal and Vascular End-Stage Disease). These are prospective, longitudinal community-based observational cohort studies [20,21,22]. These data suggest that the association between cardiovascular health and future cancer is attributable to shared risk factors [19]. The study by Zhang et al. [18] included a large population and a long follow-up, thus showing evidence that a clearer benefit is detected only after 10 years of aspirin use, and it persists despite continuing use or not. This 10-year latency before the benefit of aspirin-based chemoprevention was previously shown in several studies.

Mechanisms driving aspirin potential in CRC chemoprevention are still under debate and will be discussed below.

Overall, there is no doubt that aspirin can play a significant role in cancer prevention, especially for large bowel cancer; moreover, low-dose aspirin is recommended for the management of acute ischemic syndromes (both coronary and cerebrovascular) and for the prevention of their recurrence [10]. However, the role in the primary prevention of atherothrombosis and cancer remains controversial because of the uncertain balance of the potential benefits and risks. The main hazard of low-dose aspirin therapy is hemorrhage due to inhibition of platelet function, which is an important component of primary hemostasis. In middle-aged patients, the increased risk of bleeding corresponds to an estimated absolute excess of approximately 1–2 major bleeding complications per 1000 patients treated with low-dose aspirin for 1 year; the excess risk is smaller in young people and substantially higher in elderly individuals and in those with a history of ulcer bleeding [10].

Clinical decision-making in this setting is guided by evaluating the risk of cardiovascular disease, CRC, and bleeding [23]. The U.S. preventive services task force (USPSTF) recommends initiating low-dose (81mg/day) aspirin use for CRC primary prevention in adults aged 50–59 years or 60–69 years who have a 10% or greater 10-year cardiovascular disease risk, are not at increased risk of bleeding, have a life expectancy of at least 10 years, and are willing to take low-dose aspirin daily for at least 10 years. In contrast, evidence is insufficient among those aged 50 years old and younger or 70 years and older. Based on new analyses of the evidence from primary cardiovascular disease prevention [24] and the results of the ASPREE trial [25], the USPSTF has changed the age ranges and grades of its recommendation on aspirin use (https://www.uspreventiveservicestaskforce.org/uspstf/draft-recommendation/aspirin-use-to-prevent-cardiovascular-disease-preventive-medication#bootstrap-panel--4, accessed on 1 December 2021). The decision to initiate low-dose aspirin use for the primary prevention of cardiovascular disease in adults ages 40 to 59 years who have a 10% or greater 10-year cardiovascular disease risk should be an individual one. USPSTF recommends against initiating low-dose aspirin use for the primary prevention of cardiovascular events in adults age 60 years or older. However, persons who are not at increased risk of bleeding and are willing to take low-dose aspirin daily are more likely to benefit. The ASPREE trial put a substantial warning against using aspirin in elderly subjects [25]. The trial enrolled 19,114 persons with a median age of 74 years. Follow-up was stopped at 4.7 years since aspirin use did not improve the primary endpoint, i.e., disability-free survival (death, dementia, or persistent physical disability), and a higher rate of hemorrhagic events was obverted (HR, 1.38; 95% CI, 1.18–1.62). In particular, the overall mortality was higher in the aspirin arm (HR, 1.14; 95% CI, 1.01–1.29) with a major contribution of cancer-related mortality (HR, 1.31; 95% CI, 1.10–1.56). The message was that in the elderly, the risk-benefit ratio of primary prevention with aspirin is not advisable. Notably, cancer prevention is not realistic in an older population since a long time is needed to obtain anticancer protection.

Nevertheless, dose and duration remain open issues.

Precision medicine by tailoring drug treatment to specific individuals or populations could help to improve efficacy while reducing side effects; however, selecting the appropriate target population requires information on genetic and other biomarkers, together with environment and lifestyle for each person. These detailed patient data will be integrated using structured ontological approaches, analytics, mathematics, and statistics which constitute the tools of quantitative systems pharmacology; this strategy can predict drug efficacy and safety on an individual basis. The selection of patients and the dose for aspirin treatment will be performed using machine learning algorithms based on demographic, clinical, genetic, and biochemical information, such as BMI, diabetes, previous cardiovascular diseases, smoking status, genetic variants, inflammatory status, microenvironment (including microbiota and DNA methylation status), and risk factors for susceptibility to bleeding.

The ADD-Aspirin randomized trial [26] is ongoing and will allow achieving information on the aspirin dose to prevent recurrence and survival for colorectal, gastro-esophageal, breast, and prostate cancer. This is a three arms study for individuals <75 years (placebo, 100 mg, or 300 mg daily aspirin), and only two arms (placebo or 100 mg daily aspirin) for subjects ≥75 years old. The open-label run-in data, available for 2253 participants, are encouraging: grade 1–2 dyspepsia was the most frequent adverse event (11%), and only 0.5% of grade 3 side effects were reported.

## 3. Clinical Pharmacology of Aspirin and Non-Invasive Biomarkers for Drug Response Prediction and Monitoring

### 3.1. Mechanism of Action of Aspirin

Aspirin (i.e., acetylsalicylic acid, ASA) is an NSAID [27]. At therapeutic doses, NSAIDs act by inhibiting prostanoid biosynthesis in different cell types, therefore producing anti-inflammatory, analgesic, and antipyretic effects associated with lower incidence of some side effects (mainly bleeding and hypertension) [27]. The therapeutic actions are mediated primarily by inhibiting the COX activity of the inducible COX-2. Aspirin causes analgesic and anti-inflammatory effects when administered at 350–600 and 1200 mg, respectively [28].

In contrast to other NSAIDs that rapidly or slowly reverse inhibitors of COX-isozymes, the binding of aspirin to the COX active site is followed by covalent modification of COX-1 and COX-2, i.e., the acetylation of a specific serine residue located at position 529 and 516, respectively [27]. Thus, aspirin causes irreversible inhibition of the catalytic activity of COX-isozymes, which become unable to transform arachidonic acid (AA) to prostaglandin (PG)G_2_ and PGH_2_, the common substrate for many downstream isomerases and synthases, catalyzing its conversion to different prostanoids [27].

### 3.2. Aspirin Affects Platelet COX-1 at Low-Doses

Aspirin has a short half-life (approximately 20 min); thus, the aspirin pharmacological effect duration depends on the rate of COX-2 protein synthesis. In a nucleated cell, COX-2 turnover is fast; thus, aspirin has to be administered every 4–6 h to obtain a persistent pharmacological effect. Once-daily aspirin administration at medium-high doses can produce only a transient inhibition of COX-2 activity due to rapid de novo protein synthesis. Several lines of evidence have shown that some NSAIDs, including aspirin, can inhibit the proliferation and induce apoptosis of colon cancer cells in vitro independently from their inhibitory effect on COX-2-dependent prostanoid biosynthesis [28]. However, these off-target effects have been mainly detected in vitro at very high concentrations of aspirin, often in the millimolar range that are not reached in vivo, in the systemic circulation, even when aspirin is administered at high anti-inflammatory doses.

In contrast, the irreversible inactivation of COX-1 in platelets (which do not express COX-2) by aspirin is long-lasting due to a low rate of de novo protein synthesis in the anucleated cell. Thus, low doses (75–100 mg) of aspirin given once a day are appropriate to cause a maximal inhibitory effect of platelet COX-1 activity (almost complete), which persists throughout the interval between doses [29]. Another interesting aspect of aspirin pharmacodynamics is that the drug has an early inhibitory action on platelet COX-1 in the presystemic circulation before first-pass partial deacetylation by the liver [30]. The peculiar pharmacodynamics of low-dose aspirin explains its improved gastrointestinal safety versus high-dose aspirin or non-aspirin NSAIDs [31].

Within the limitations of post hoc analyses of cancer events that were not pre-specified endpoints, the results of the Thrombosis Prevention Trial are of interest to make a mechanistic interpretation since the anticancer effect of aspirin was apparent in men at high cardiovascular risk treated with a 75-mg controlled-release aspirin formulation associated with very low concentrations (approx. 0.29 μM) measured in the systemic circulation [32]. This aspirin formulation was developed to maximize cumulative inhibition of platelet COX-1 in the pre-hepatic circulation and minimize inhibition of COX-2 in the systemic compartment [33]. Thus, the drug acts by inhibiting selectively platelet function with a limited systemic effect. Moreover, reduced risk of CRC (a pre-specified secondary endpoint) was detected in the long-term observational follow-up of the Women’s Health Study where aspirin was administered in alternate-day 100-mg aspirin versus placebo [34]. These findings suggest that the chemopreventive effect of aspirin recapitulates its antiplatelet effect, i.e., a long-lasting duration [29] and, most importantly, its saturability at low doses [35].

Platelets are activated in response to environmental factors, atherosclerotic plaque rupture or fissuring, and intestinal mucosa damage. Activated platelets release a vast repertoire of mediators that may evoke numerous signaling pathways associated with a phenotypic switch of the cellular compartment of the stromal environment [36]. These events alter epithelial and stromal cell interactions and create a tissue microenvironment that promotes intestinal neoplasia. A key event is represented by enhanced biosynthesis of prostaglandin (PG)E_2_ in the intestinal mucosa, which occurs in the early stages of tumor development through cyclooxygenase (COX)-1 activity, in association with the suppression of the prostaglandin-degrading enzyme 15-prostaglandin dehydrogenase (15-PGDH) [6]. Later, COX-2 is induced and further increases PGE_2_ production, thus promoting colorectal adenoma [37] and its progression to adenocarcinoma [38].

### 3.3. Low-Dose Aspirin Causes a Direct Inhibitory Effect on COX-1 Expressed in Colorectal Mucosa

The development of a novel direct biomarker of drug action via the assessment of the acetylation of COX-1 at serine 529 has allowed showing that low-dose aspirin acetylates normal colorectal mucosal COX-1 [29], even if at a lower extent versus platelet COX-1 [39]. This effect of low-dose aspirin is associated with reduced biosynthesis of colorectal mucosal COX-1-dependent PGE_2_ and its capacity to induce the phosphorylation of ribosomal protein S6 (p-S6) [39]; p-S6 regulates the size of some cell types but is also dispensable for the translational control of mRNAs.

The ratio of p-S6/S6 is associated with tumor progression. Thus, the antitumorigenic effect of low-dose aspirin can also involve the prevention of S6 phosphorylation via the inhibition of colorectal mucosal COX-1-dependent PGE_2_ generation.

Enhanced PGE_2_ production can also disrupt the normal apoptotic processes, allow the affected cells to accumulate genetic mutations, and ultimately lead to loss of proliferative control [37,38].

Moreover, it may suppress immune functions and facilitate tumor immune escape.

Thus, low-dose aspirin can affect early events of intestinal tumorigenesis indirectly via the inhibition of platelet function and in part directly by affecting intestinal mucosa COX-1-dependent PGE_2_.

### 3.4. Low-Dose Aspirin Can Indirectly Prevent COX-2 Induction in Colorectal Mucosa

The induction of COX-2 in the colorectal mucosa is associated with the aberrant increase of PGE_2_ production, thus promoting the progression of colorectal adenoma to adenocarcinoma [6]. The possible contribution of platelet activation on COX-2 induction first in the stromal compartment and then in the epithelial cells of the colorectum has been proposed [6]. Low-dose aspirin can indirectly prevent COX-2 induction by constraining platelet function. Whether low-dose aspirin can also directly inhibit colorectal COX-2 activity and PGE_2_ biosynthesis remains to be demonstrated. It is still unknown whether higher doses of aspirin can translate into a more profound inhibitory effect COX-2, thus improving the anticancer effect. Mechanistic studies are ongoing in Patrignani’s laboratory to address these issues, using the recently developed aspirin biomarker, which assesses the extent of acetylated COX-2 at serine 516 in CRC tissues together with the assessment of prostanoid biosynthesis in the cancer biopsies of CRC patients treated with aspirin at low and medium doses [40].

### 3.5. Assessment of Biomarkers to Develop a Precision Therapy of CRC with Aspirin

Platelets can uptake circulating proteins and RNAs/microRNAs, thus acquiring a different intracellular molecular repertoire specific to the individual clinical condition. The analysis of platelet content (and of platelet-derived microvesicles), as a whole of transcript (transcriptomics) or proteins (proteomics), has the promise of being a novel tool for diagnosis and prognosis of several diseases, including cancer [41].

The analysis of systemic prostanoid biosynthesis (by measuring the urinary levels of prostanoid enzymatic metabolites) in patients at baseline or after aspirin treatment provides important information about the impact of aspirin on platelet activation, vascular and inflammatory state [29,39,42].

Plasma markers, such as soluble tumor necrosis factor receptor-2, as well as tumor expression levels of genes involved in prostanoid biosynthesis or signaling pathways activated by the aberrant expression of COX-2, such as phosphatidylinositol 3-kinase [43,44] have been suggested to identify those subjects who will respond to the antineoplastic effect of aspirin. Most of these studies suffer from the limitation of investigating large cohorts of nonrandomized participants who provided data on aspirin use in a questionnaire. Thus, these findings should be confirmed by large randomized clinical trials.

A systems biology approach to the analysis of heterogeneous datasets (genomics, epigenomics, proteomics, lipidomics, and clinical) would allow dynamic systems modeling of candidate pathways involved in the antineoplastic effect of aspirin. This strategy would also allow identifying susceptibility profiles for CRC and their use to develop new biomarkers to predict its occurrence and recurrence.

## 4. Cancer Prevention in Lynch Syndrome Carriers

Lynch syndrome, caused by germline mutations of DNA mismatch repair (MMR) genes, accounts for about 3.0–5.0% of all CRCs; carriers of pathogenic variants face a CRC lifetime risk of approximately 50% (considering *MLH1* and *MSH2*, lower for the other syndromic genes) [45]. For this reason, intensive endoscopic surveillance is recommended, but it seems that endoscopy reduces CRC mortality without decreasing incidence in this population [46]. This observation is consistent with the innovative theory of three pathways for CRC in this setting [47,48]: (1) A subset of Lynch syndrome CRCs develops through MMR deficiency-independent adenoma formation with secondary MMR inactivation; most commonly, however, tumor formation follows or is initiated by MMR deficiency, which can either lead to (2) MMR-deficient adenoma formation, or to (3) entirely nonpolyposis progression into invasive cancer.

Lynch syndrome carriers are a very high-risk population, appropriate for cancer prevention trials where aspirin may play as an “adjuvant therapy” to improve surveillance results (but not to quit colonoscopy).

The CAPP2 trial [49] was designed to investigate the antineoplastic effects of aspirin (at 600 mg/day) and resistant starch in Lynch syndrome carriers. The study recruited 1009 patients with an MMR pathogenic variant, the median age was relatively young (43–44 years old), and the median time of aspirin intake was 26.5 months. A first publication [49], at intervention completion, reported no beneficial effect in the aspirin arm nor the starch resistant arm. To note no excess of serious adverse events was reported in the aspirin arm.

A planned 10 year follow-up publication [50] showed a non-significant aspirin preventive effect in the intention-to-treat analysis (HR = 0.63, 95% CI, 0.35–1.13), but a significant 59% CRC reduction in subjects who took aspirin regularly for at least two years (HR = 0.41, 95% CI, 0.19–0.86; *p* = 0.02) with a similar prevention rate also for all other syndromic cancers.

With 20 years of follow-up [51], intention-to-treat analysis specifically for CRC showed a significant 35% risk reduction (HR = 0.65, 95% CI, 0.43–0.97; *p* = 0.035), and this result was significantly evident in overweight patients. The protective effect for extra-colonic cancers seen in the previous publication was not confirmed even in the compliant subjects (HR = 0.75, 0.42–1.34). No significant differences of adverse events (bleeding, gastrointestinal disease) in aspirin subjects vs. placebo were observed. This finding is important to support the use of aspirin since adverse events are still the major barrier to its clinical use in primary prevention.

Similarly, to the data obtained from the cardiovascular trials [8,9], the CAPP2 trial shows the delay of aspirin efficacy, as the cancer incidence started to separate between the two arms after five years, but even more important is that a relatively short treatment (median 26.5 months) provided such a long-lasting effect. Furthermore, since the preventive effect was statistically significant also in the intention-to-treat analysis, thus the aspirin preventive effect has been strengthened.

### Guidelines and Recommendations

Table 1 summarizes some of the relevant international guidelines in CRC prevention medicine. For sporadic CRC, there are still debates among groups as to whether aspirin should be considered for primary prevention. Whereas, for LS carriers there is general agreement that the evidence of aspirin efficacy is strong enough to consider and discuss its use within this high-risk population, even with the remaining doubts about dosage and duration. Although the CAPP2 results are based on a treatment of 600 mg per day (300 mg/BID), the dose of 81–100 mg per day is suggested by some groups without necessarily waiting for the results of the CAPP3 study [52].

Among cancer prevention guidelines, the Cancer Council Australia (CCA) in 2017 updated its indication for CRC prevention, suggesting that all people aged 50–70 years with an average risk of CRC should consider taking low-dose aspirin to reduce their CRC risk (Clinical Guidelines Wiki 2019 [53]). Regardless of age, a similar level of evidence can be considered for subjects with an increased CRC risk due to personal or familial history. A greater level of evidence is confirmed for subjects with Lynch Syndrome, who may start aspirin treatment at the first colonoscopy [53].

Since the guideline publication, there has been no widespread modification in CRC prevention in clinical practice. Ongoing studies have been launched to better understand the awareness of physicians and the general population of CRC prevention, particularly the opinions and obstacles to taking aspirin [54,55].

As reported above, the United States Preventive Services Task Force considers taking aspirin for prevention based on personal cardiovascular risk level (10% or greater at 10 years) with an age range from 50 to 69 years old [23]. Within this population, aspirin treatment between 50 and 59 years old can translate into CRC prevention due to the latency of the aspirin effect. The guideline does not specifically consider CRC risk categories. For this aspect, the persons should refer to their general practitioner [23].

In the United Kingdom, the National Institute for Health and Care Excellence (NICE) and the British Society of Gastroenterology (BSG), together with the Association of Coloproctology of Great Britain and Ireland (ACPGBI) and the United Kingdom Cancer Genetics Group (UKCGG) guidelines specifically for Lynch syndrome carriers, recommend considering aspirin intervention for at least two years [56,57].

The NCCN guidelines, probably one the most popular and frequently mentioned publications, recommend in this regard the use of aspirin but in a personalized way, in that the decision should be made on an individual basis (as well as the dose) after a discussion in which all aspects including individual risk profile, benefits, and side effects are fully considered.

Same indications for Lynch Syndrome carriers are reported in the European Hereditary Tumor Group (EHTG) guidelines and the European Society of Coloproctology (ESCP). Their indication is to use aspirin at low doses (75–100 mg daily) and consider a higher dose for overweight subjects [58].

In the decision process to aspirin use for CRC prevention in LS carriers, several issues have to be considered to balance pros and cons. In Table 2 we summarized the main point to consider: (a) Age: older age is a contra since toxicity is increased and cancer prevention benefit requires years to be effective; (b) Higher risk genotypes (*MLH1, MSH2, MSH6*) could benefit more from a preventive intervention than *PMS2*; genotype may also influence the starting age: for *MLH1* and *MSH2* carriers the starting age could be 25 years old since their cumulative risk to develop cancer reaches 10% at 35 years, whereas MSH6 carriers could start aspirin later, approximately at 35/40 years old, since their cancer risk reaches 10% at 60 years old, and even later, if any *PMS2* carriers [59]; (c) For fertile women, the therapeutic advice has to be decided accordingly with their familial reproductive plan; (d) Helicobacter pylori should be tested and, if positive, eradicated before starting aspirin treatment; (e) a concomitant cardiovascular disease increased risk, a previous diagnosis of CRC with a preserved portion of colon-rectum, or any other syndromic cancer with no sign of recurrence and an overall good prognosis are in favor of aspirin use; (f) BMI is controversial since in the CAPP2 study higher BMI was associated with an increased aspirin benefit [60], whereas other data suggest that aspirin efficacy could be reduced by high BMI [61]; excess body fat is a modifiable cancer risk factor [62], and platelet activation is associated with obesity [63]; thus, aspirin might be beneficial to cancer patients with obesity; however, it was reported that obesity affects aspirin metabolism in several ways that reduce its inhibitory effect on platelet function [64]; clinical studies are ongoing by Patrignani’s group to verify whether aspirin antiplatelet effects could be improved by increasing the aspirin dose or shortening its dosing interval in CRC patients with obesity.

## 5. Conclusions

We intend to discuss the information on aspirin as preventive medicine with all LS carriers followed by all the AIFET (Associazione Italiana Familiarità Ereditarietà Tumori, former AIFEG) adhering members. In line with the major international guidelines, the proposed dose will be 100 mg per day. The results from sporadic CRC prevention frame [32,34] together with the aspirin pharmacodynamics data [29] support that the effect for cancer prevention can be saturable at low doses. The gold standard should be to wait for the results of the CAPP3 study to clarify the best dose to use, but they will not be available for several years (expected end of recruitment 2024). With the already available data for CRC prevention, the general opinion is that this high-risk population should also be informed of the aspirin preventive effects although with the remaining open question on the dosage. The intervention should be taken for a minimum of two years. The decision to start aspirin treatment has to be taken after an adequate discussion to identify the individuals whom can gain the most benefit and are less likely to experience adverse events due to aspirin intake.

In conclusion, we propose a spontaneous program within the AIFET network (former AIFEG), discussing with Lynch Syndrome carriers the possibility of taking aspirin for CRC prevention and, after acceptance, monitoring for treatment adherence and side effects. Our aim is also to seek predictive surrogate biomarkers and to monitor treatment efficacy.

## Figures and Tables

**Table 1:** A schematic representation of the main guideline recommendations for colorectal cancer preventive medicine with aspirin.

Organization	Reccomandations
United States Preventive Services Task Force	Adults aged 50–69 years with 10 years CVD ≥10% * Low-dose aspirin (81–100 mg daily) Treatment for 5 to 10 years
Cancer Council Australia	People aged 50–70 years with average risk of colorectal cancer ** Dose from 100 to 300 mg daily Lynch Syndrome carriers aged ≥25 years Dose from 100 to 600 mg daily Treatment for at least 2.5 years
National Institute for Health and Care Excellence (NICE guideline)	People with Lynch syndrome Treatment for more than 2 years
BSG/ACPGBI/UKCGG	Lynch Syndrome carriers 100 mg per daily ***
EHTG/ESCP	Lynch Syndrome carriers 75–100 mg daily

* For the age range 60–69 has to consider the latency of 10 years before to lower colorectal cancer risk, together the other variables. ** The decision to start the treatment should be personalized based on: age and life expectancies since the benefit is evident after 10 years; sex male may have longer beneficial effect; parallel reduction in cardiovascular events; concomitant risk of aspirin side effects. *** Higher dose for overweight individuals.

**Table 2:** Schema of Pros and Cons to consider for aspirin as cancer prevention agent in LS carriers.

Variable	Pros	Cons
Age	<60 years old	≥60 years old
Genotype	MLH1 MSH2 MSH6 (PMS2 *)	
Sex	M/F	Child bearing or desire of pregnancy
Allergy	No	Known NSAIDs ipersensibility
Helicobacter Pylori	Negative	HP positive
Cancer history **	Previous syndromic cancer including CRC	protocolectomy
Cardiovascular risk ***	≥10%	--

* *PMS2* is opinioable for is moderate risk; ** Cancer disease free survival ≥ 5 years and non-evidence of recurrences; *** based on available algorithms like Framingham Risk Score.

## Data Availability

Not applicable.

## Notes

*This paper has been developed out of a round table at the “XVIII Congresso Associazione Italiana per lo Studio della Familiarità ed Ereditarietà dei Tumori Gastrointestinali (A.I.F.E.G., now A.I.F.E.T. Associazione Italiana Famigliarità ed Ereditarietà Tumori)” October 2021.*
